# A Fall Risk mHealth App for Older Adults: Development and Usability Study

**DOI:** 10.2196/11569

**Published:** 2018-11-20

**Authors:** Katherine L Hsieh, Jason T Fanning, Wendy A Rogers, Tyler A Wood, Jacob J Sosnoff

**Affiliations:** 1 Department of Kinesiology and Community Health University of Illinois at Urbana Champaign Urbana, IL United States; 2 Department of Internal Medicine Wake Forest School of Medicine Winston-Salem, NC United States

**Keywords:** usability, fall risk, mHealth app, mobile phone

## Abstract

**Background:**

Falls are the leading cause of injury-related death in older adults. Due to various constraints, objective fall risk screening is seldom performed in clinical settings. Smartphones offer a high potential to provide fall risk screening for older adults in home settings. However, there is limited understanding of whether smartphone technology for falls screening is usable by older adults who present age-related changes in perceptual, cognitive, and motor capabilities.

**Objective:**

The aims of this study were to develop a fall risk mobile health (mHealth) app and to determine the usability of the fall risk app in healthy, older adults.

**Methods:**

A fall risk app was developed that consists of a health history questionnaire and 5 progressively challenging mobility tasks to measure individual fall risk. An iterative design-evaluation process of semistructured interviews was performed to determine the usability of the app on a smartphone and tablet. Participants also completed a Systematic Usability Scale (SUS). In the first round of interviews, 6 older adults participated, and in the second round, 5 older adults participated. Interviews were videotaped and transcribed, and the data were coded to create themes. Average SUS scores were calculated for the smartphone and tablet.

**Results:**

There were 2 themes identified from the first round of interviews, related to perceived ease of use and perceived usefulness. While instructions for the balance tasks were difficult to understand, participants found it beneficial to learn about their risk for falls, found the app easy to follow, and reported confidence in using the app on their own. Modifications were made to the app, and following the second round of interviews, participants reported high ease of use and usefulness in learning about their risk of falling. Few differences were reported between using a smartphone or tablet. Average SUS scores ranged from 79 to 84.

**Conclusions:**

Our fall risk app was found to be highly usable by older adults as reported from interviews and high scores on the SUS. When designing a mHealth app for older adults, developers should include clear and simple instructions and preventative strategies to improve health. Furthermore, if the design accommodates for age-related sensory changes, smartphones can be as effective as tablets. A mobile app to assess fall risk has the potential to be used in home settings by older adults.

## Introduction

In adults 65 years or older, 1 in 4 will fall per year [1]. Falls are also the leading cause of injury-related death in older adults [2]. The Centers for Disease Control and Prevention recommends annual falls screening for all older adults. However, objective fall screening is rarely assessed in clinical settings, in part because it requires expensive equipment, clinicians have time constraints, or they may not have the training or relevant expertise [3].

Mobile technology such as smartphones offer a potential solution for measuring fall risk objectively, inexpensively, and with minimal training required. Unlike gold-standard technology for fall risk assessment (ie, force plates, stand-alone accelerometers, and high-speed motion capture cameras), smartphones are commercially available and cost-efficient [4]. While smartphone technology may be useful, older adults tend to be slow to adopt new technologies [5]. However, having high perceived usefulness (the belief that using a system enhances performance) and high perceived ease of use (the belief that a system is free of effort) are foundational determinants of technology acceptance [6].

High perceived usefulness and perceived ease of use may explain why older adults are the fastest-growing population of smartphone users [7]. As of 2017, 42% of adults 65 years and older own a smartphone [8]. Moreover, 74% of adults aged 50-64 years own smartphones [8]. Furthermore, the use of health apps is growing among older adults, enabling these individuals to monitor and improve their health through their own device [9]. In addition to tracking health, apps can also provide feedback to help users reach health goals [10]. Therefore, the adoption of smartphone apps for older adults should be designed for high perceived ease of use and perceived usefulness.

There is growing evidence of the validity of smartphone usage for fall risk screening. There have been 2 recent systematic reviews that have indicated that smartphone accelerometers and smartphone apps have the potential to measure fall risk through quantifying gait and balance [11,12]. For instance, Ozinga and Alberts [13] found high correlations in root mean square acceleration and 95% volume of acceleration between an iPad and 3D-motion capture during static balance conditions. An app called the uTUG, which measures performance during the Timed Up and Go (TUG), was developed by 1 group [14]. Additionally, another study developed a fall risk app based on the Aachen Falls Prevention Scale and found their app to be related to users’ self-reported history of falls [15]. The growing body of evidence for smartphone use to measure fall risk brings strong potential for falls screening in the home setting. This provides an opportunity for older adults to assess their individual fall risk, a necessary step in determining the type of fall prevention treatment needed.

Health apps targeting falls prevention are becoming more common, but previously developed apps center on a single task, such as the TUG or a specific questionnaire. Because fall risk is multifactorial, there is a need for an app to assess multiple influences on fall risk. These past apps provide an important foundation for mobile fall risk assessment, but an app that measures multiple risk factors helps determine which measures are useful for individualized fall risk. In addition to developing health apps to measure fall risk, a critical next step is to ensure usability by the target users. Our review of the literature indicated that the usability aspect is typically not part of the reported evaluations [13,16,17]. A smartphone can effectively address the issue of inadequate fall risk screening but only if older adults are able to use the app. A usable app must be designed to accommodate age-related changes in perceptual, cognitive, and motor capabilities [18]. Designing for accommodation of age-related changes is necessary but not sufficient for ensuring usability by older adults [19]. Usability testing with members of the target user group is needed to identify additional usability challenges. Therefore, the purpose of this study was to develop a fall risk app and test the usability of the app in healthy, older adults. A smartphone app that measures fall risk through a battery of assessments and is usable by our target population will improve falls screening and help identify those in need of fall prevention resources.

## Methods

### App Design and Development

**Figure 1:**
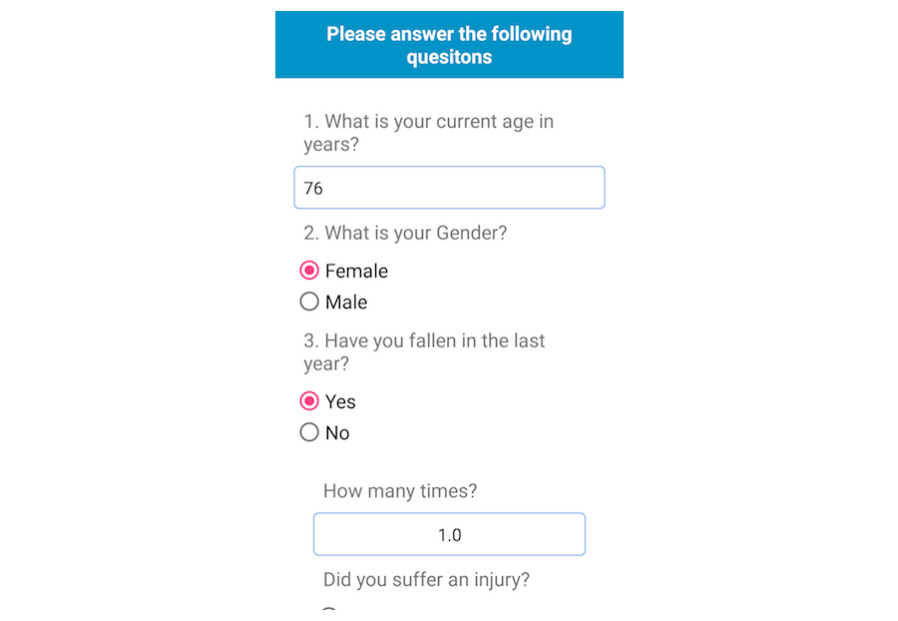
Screenshot of the healthy history questionnaire.

**Figure 2:**
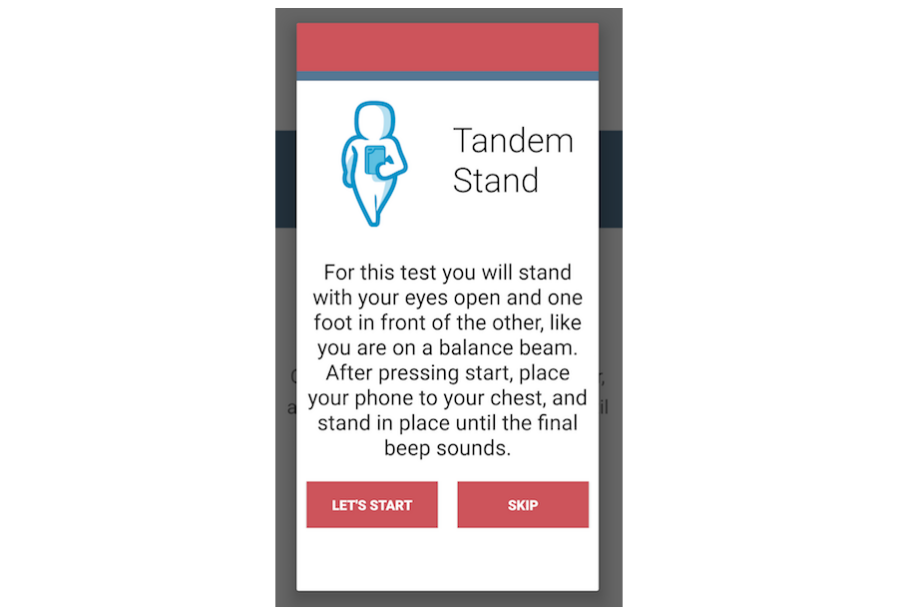
Screenshot of the tandem stance task.

**Figure 3:**
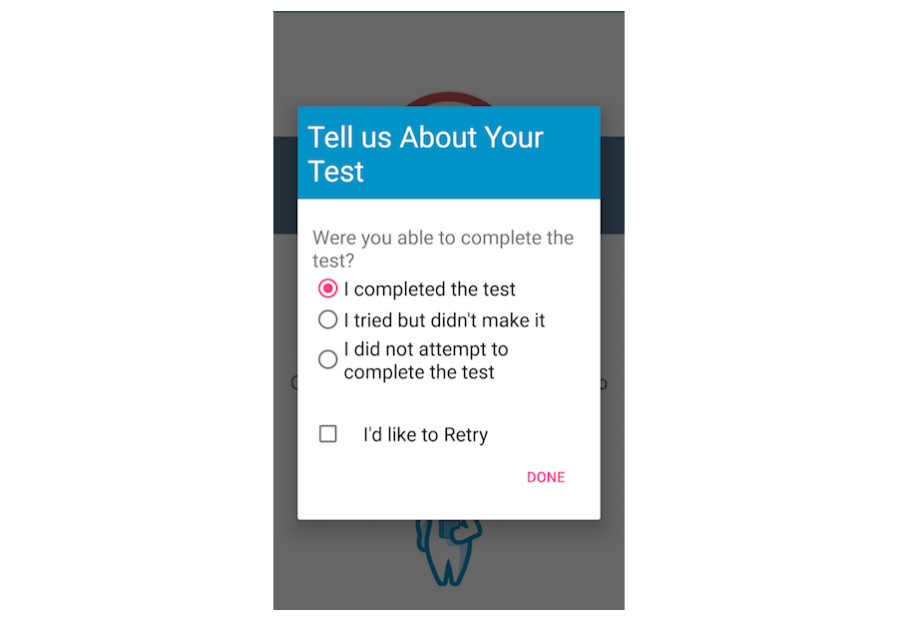
Participants report their ability to complete each balance task.

**Figure 4:**
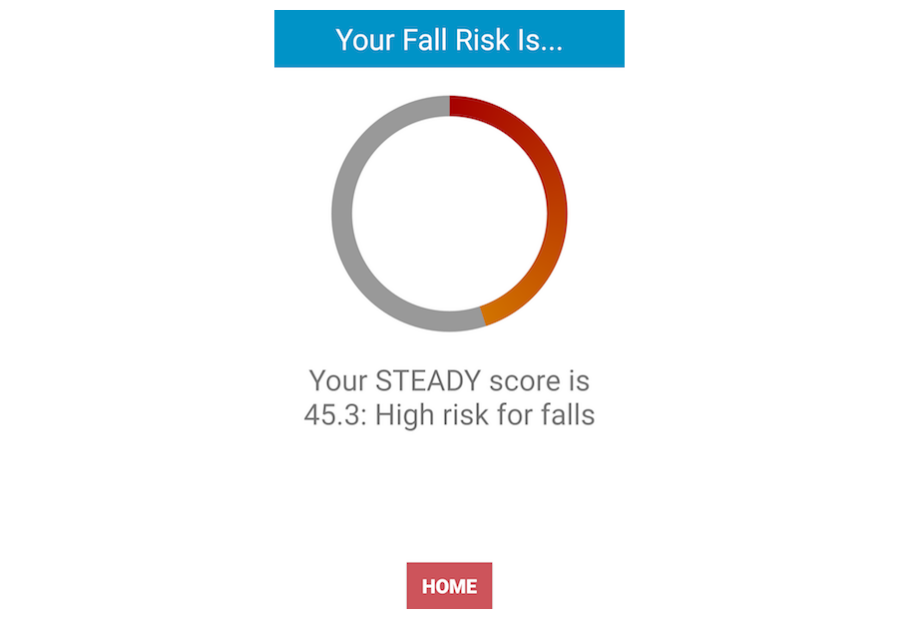
Final fall risk score; lower scores represent a greater risk for falls.

The fall risk app, Steady, was developed in Android Studio 3.1.2 for smartphone and tablet devices. Steady consists of 2 components. The first is a 13-item questionnaire of health history (ie, age, gender, number of falls in the last year, and perceived balance confidence [20]; Figure 1). These questions were chosen because they are associated with falls and recurrent falls in community-dwelling older adults [21]. The second component is a progressive postural stability test (Figure 2), wherein the device guides participants through 5 progressively difficult tasks. These include 4 30-second balance tasks (eyes open, eyes closed, tandem, and single leg) plus a 30-second sit-to-stand test. These tasks were chosen because they have been shown to discriminate between high and low risk of falling in older adults [4,22]. Instructions prior to each balance task are provided through text, and users are asked to hold the phone against their chest for the duration of each test. Safety instructions to wear sturdy shoes and the option to skip a balance task were provided prior to the start of each task. On completion of each task, users report whether they attempted the task. If so, they then report whether they were able to complete the task (Figure 3). These data, alongside data from the health history questionnaire, are entered into a weighted algorithm to produce a score ranging from 0-100 and classified into a very low, low, moderate, high, or very high risk of falling (Figure 4). Lower scores represent a greater risk for falls.

The first iteration of the app was developed with consideration for age-related changes that might influence usability. With respect to sensory changes, we ensured that the font was at least size 14 and sans serif, the recommended font size and type for older adults [23]. All text is black text on a white background to maximize contrast. The app also utilizes an audio component for the balance tasks. To begin each balance task, 5 identical beeps are presented, and the 30-second posture task begins at a unique (ie, higher) sixth beep. We ensured that the audio is loud enough for older adults to hear, and we added vibrations during each auditory tone for those hard of hearing. We minimized the cognitive demands by listing 1 set of instructions or task per screen. In doing so, we aimed to reduce working memory overload. In total, there are 10 screens to navigate before receiving a final fall risk score. Lastly, we accommodated motor capabilities of older adults by incorporating large buttons when entering information.

### Usability Testing

#### Participant Characteristics

A total of 11 healthy, older adults participated in usability testing. Our approach was to have older adults interact with the app, identify usability issues, improve the design, and then have another group of older adults interact with the app. This iterative approach is ideal for identifying use challenges. Nielson [24] has argued that small numbers of participants (~5) are sufficient for identifying usability problems. Consequently, we included 6 older adults in our first iteration and 5 in our second. Inclusion criteria included being over 70 years of age, self-reported ability to swipe on a touchscreen device, and able to stand with or without aid. All procedures were approved by the University of Illinois at Urbana-Champaign Institutional Review Board, and all participants completed written informed consent prior to participation.

#### Testing Environment

Usability testing was performed at 2 sites selected for convenience for the participants. The first was an unoccupied apartment at a local retirement community. Testing was performed in the living room of this apartment to mimic using the app in one’s own home. We tested 5 participants from the first iteration and 3 participants from the second iteration at the apartment. The second site was a research laboratory on a University campus. This site used an open space in the research laboratory. We tested 1 participant from the first iteration and 2 participants from the second iteration at the research lab.

#### Procedures

An iterative design-evaluation process of semistructured, videotaped interviews was used to determine the optimal usability of Steady [25]. Participants were presented with a smartphone (Samsung Galaxy S6) and tablet (Samsung Galaxy Tab S3) and asked to pick their choice of device to use first. After selecting a device, participants were asked to open the app and follow all instructions while thinking aloud and narrating their thoughts. In addition, a series of open-ended questions related to ease of use, recommendations for modifications, and feasibility were asked after completing the healthy history questionnaire, after completing the balance tasks, and after receiving their final fall risk score. These questions are included in Multimedia Appendix 1. After completion, participants repeated the testing measures with the alternate device and were asked to report differences in using the smartphone and tablet. Participants then completed the Systematic Usability Scale (SUS) [26] for both the smartphone and tablet. The SUS is widely used to quantify the usability of user-machine interfaces, consisting of 10 standard questions on a 5-point Likert scale, with higher scores indicating greater usability [26]. The SUS ranges from 0 to 100, with higher scores representing greater usability.

After the first iterative cycle, changes were made to the app based on identified issues from the interviews. The second iterative cycle was conducted on 5 older adults, following the same format as the first cycle. No new usability challenge themes emerged after the second cycle.

#### Data and Statistical Analysis

All videotapes and field notes taken during the interview were transcribed verbatim. Qualitative data from transcripts and field notes were reviewed by KH to develop a coding system. Based on their content, data were assigned with codes, and codes with similar content were grouped into thematic categories.

Following a mixed-methods approach, SUS data were used to complement the qualitative results. SUS scores were averaged for each participant and transformed into a usability score out of 100, where the average score was 68 [27].

## Results

### Participant Characteristics

Demographic information of all participants is provided in Table 1.

### Iteration 1

#### Usability Testing Interviews

Transcript analysis and coding revealed 2 distinct usability themes, namely, perceived ease of use and perceived usefulness.

##### Perceived Ease of Use

**Figure 5:**
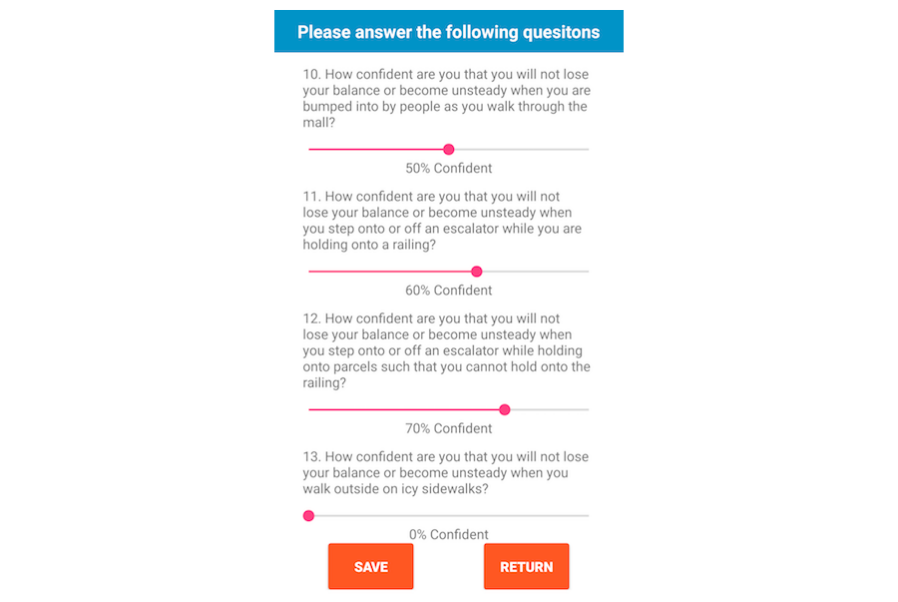
In the first iteration, balance confidence was presented as a slide bar.

**Figure 6:**
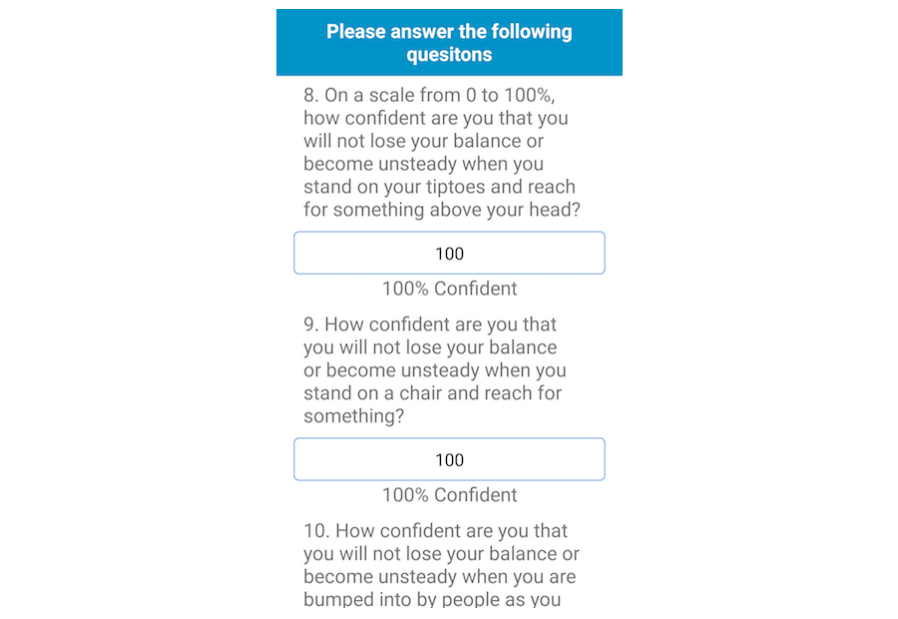
In the second iteration, the balance confidence questions were changed to key-in entries.

Overall, participants found the app easy to follow and free of clutter. Some participants found difficulties swiping between screens and answering the balance confidence questions in the health history questionnaire. In the first iteration, a slide bar was used to indicate a percentage for 0 through 100 (Figure 5). However, 4 participants were unable to drag the slide bar or needed multiple attempts. Therefore, the slide bar was replaced with a key-in entry (Figure 6), and forward and back arrows were added to each screen.

**Figure 7:**
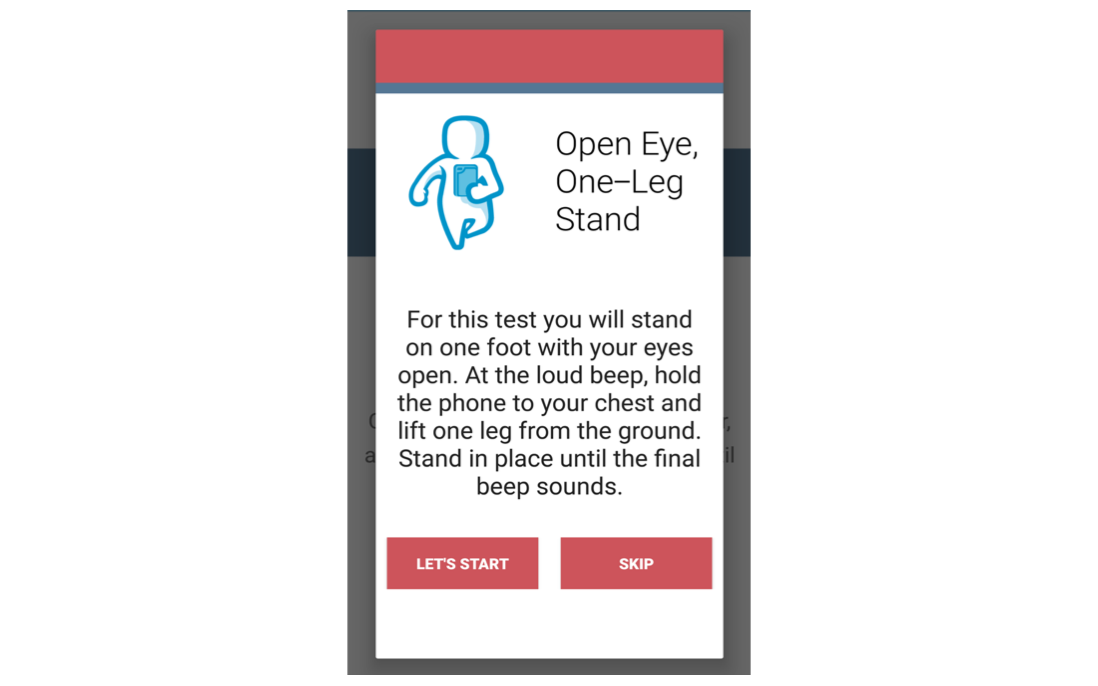
Instructions were added in the second iteration to clarify the start and end of each balance task.

Participants reported confusion following the instructions to begin and end each balance task. The first 5 beeps prior to starting each balance task was programmed to allow time for participants set-up for each task. However, 4 of the 6 participants were confused about when to start or stop each task. Participants thought the task ended at the sixth beep instead of starting at the sixth beep. As a potential solution, instructions were added prior to each balance task explaining when each task begins and ends within the second iteration of the app (Figure 7).

**Figure 8:**
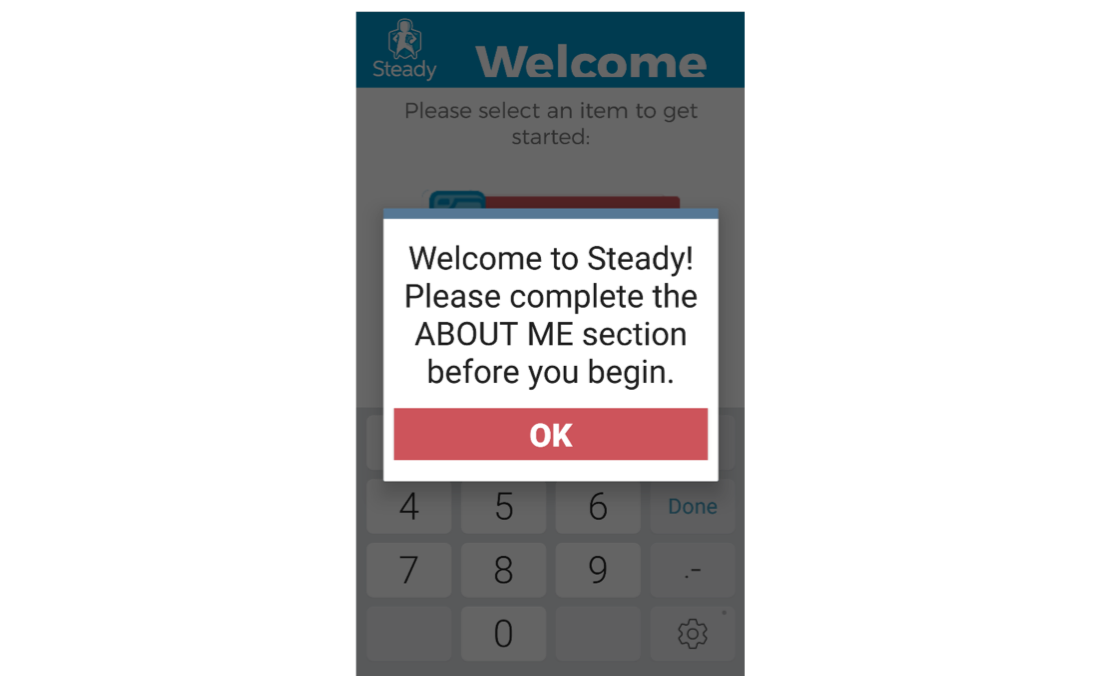
To prevent users from receiving an inaccurate fall risk score, users are prompted to answer health history questions at start up.

Additionally, participants completed the health history questionnaire and balance tasks in the incorrect order. In the first iteration, participants’ fall risk would be displayed following the completion of the balance tasks, regardless of whether the health history questionnaire was completed. This resulted in an inaccurate initial score. This resulted because the button to initiate the balance tasks was displayed above the button for the health history, and participants often completed the balance tasks first. To address this issue in the second iteration, only the questionnaire button is displayed until the questionnaire is complete (Figure 8).

In comparing using the smartphone and the tablet, there were few differences reported. No differences between the 2 devices were reported by 3 participants. There was 1 participant who preferred holding the tablet for the balance tasks, and 2 participants preferred holding the phone for the balance tasks.

##### Perceived Usefulness

Participants enjoyed learning their risk of falling from the app. There were 4 of the 6 participants who reported concerns and fear of falling, indicating that this app may address their concerns. For example, 1 participant mentioned that it helped her think about her balance and falls.

I think this [the app] would be helpful for me because I wasn’t too steady. This helps me think about balance exercises when I go to the gym.

Female, 74 years old

It appeared that the greatest benefit for participants was to receive their fall risk and be more aware of falls. Half of the participants reported this benefit. Moreover, all participants who received a high risk of falls wanted to receive fall prevention strategies to lower their fall risk.

Participants also reported confidence and acceptance in using the app on their own if it were downloaded on their own device. There were 4 participants who explained that the app was easy to follow on their own. There was 1 participant who indicated that she may need assistance to start the app but could finish on her own. While a caregiver may not be needed, some participants mentioned they would want to be near a sturdy object in case they lose their balance. This was further included in the safety instructions provided at the start of each in-app testing session.

I am comfortable using [the app] by myself.

Female, 77 years old

A summary of the main issues, with sample quotes, in the first iteration and the solutions implemented in the second iteration are described in Table 2.

#### Usability Testing Questionnaire

**Figure 9:**
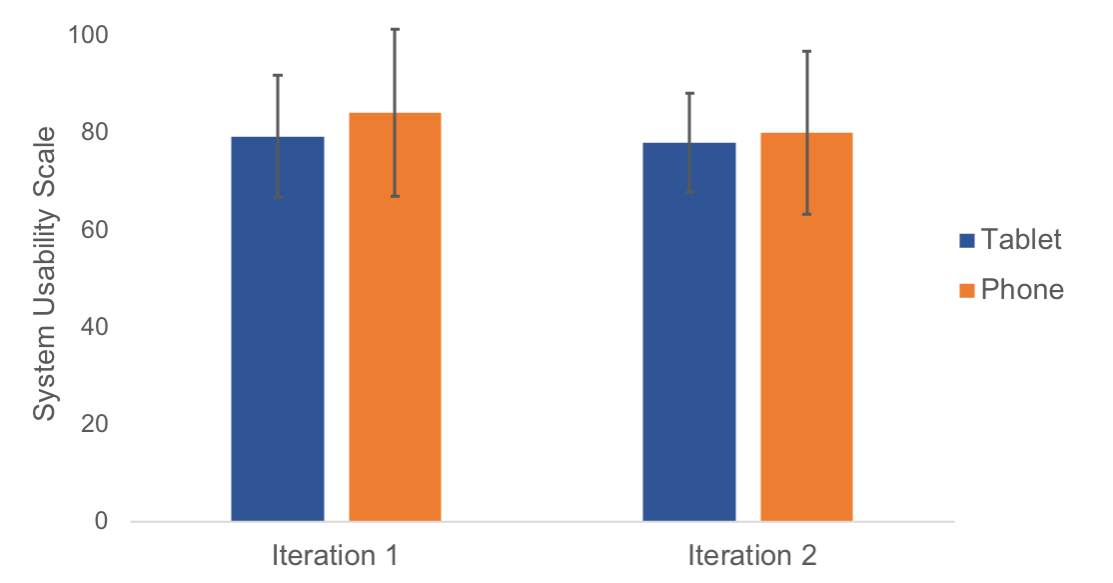
Systematic Usability Scale scores for iterations 1 and 2.

The average score on the SUS for the smartphone was 79.17, and the average score on the SUS for the tablet was 77.92 (Figure 9). Blue bars represent scores for the tablet, and orange bars represent scores for the phone. These scores represent good usability for both devices [27]. Average scores on each of the 10 items are reported in Table 3. A 5-point Likert scale was used, with higher scores indicating greater usability.

**Table 1:** Demographic information of all participants.

Characteristics		Iteration 1 (n=6)	Iteration 2 (n=5)
Age (years), mean (SD)		78.3 (7.3)	81 (3.7)
**Gender, n (%)**			
	Males	1 (17)	1 (20)
	Females	5 (83)	4 (80)
**Education, n (%)**			
	High school diploma	N/A^a^	3 (60)
	Bachelor’s degree	1 (17)	N/A
	Master’s degree	2 (33)	2 (40)
	PhD	3 (50)	N/A
Smartphone usage, n (%)		5 (83)	4 (80)
Tablet usage, n (%)		3 (50)	3 (60)
Falls in past year, range (median)		0-5 (2)	0-2 (0)

^a^N/A: not applicable.

**Table 2:** Main issues, with sample quotes, identified from the first round of usability testing with solutions implemented in the second iteration of the app.

Domain and issue		Sample quotes	Solution
**Perceived ease of use**			
	Instructions for beginning each balance task	“The instructions should be in the beginning and say the first 5 beeps do the purpose to adjust the device. Then the sixth beep is when you start the test and you do it until a final beep after 30 seconds.”	Within the second iteration of the app, instructions were added prior to each balance task explaining when each task begins and ends.
	Inaccurate fall risk score	“What do I do next?” “Do I go to Full Test?”	Only the questionnaire button is displayed until the questionnaire is complete. The Full Test button appears after the questionnaire is complete.
	Dragging a slide bar	“How do I change the number?” “My fingers can’t move the bar.”	The slide bar was replaced with a key-in entry.
	Swiping between screens	“You should indicate if I need to swipe left to right or up to down.”	Forward and back arrows were added to each screen.
**Perceived usefulness**			
	Fall prevention strategies	“It was very beneficial to get your prediction for falling. I would, at the very end, provide a link to demonstrate preventative measures to reduce the risk of falling.”	N/A^a^

^a^N/A: not applicable.

**Table 3:** Average scores on each question of the Systematic Usability Scale for iterations 1 and 2.

Item number	Smartphone		Tablet	
	Iteration 1	Iteration 2	Iteration 1	Iteration 2
1	2.4	3.3	1.6	3.2
2	2.0	1.5	2	1.5
3	4.4	4.7	4.6	4.3
4	1.2	1.3	1.2	1.5
5	3.2	4.5	3.8	4.7
6	2.2	1.3	1.4	1.3
7	4.6	4.5	4.2	4.5
8	1.2	1.2	2.2	1.5
9	4.4	4.5	4.6	4.3
10	2.0	2.0	2.0	2.3

### Iteration 2

#### Usability Testing Interviews

Transcript analysis and codes in the second round of interviews revealed 2 distinct themes, namely, perceived ease of use and perceived usefulness.

##### Perceived Ease of Use

In the second round of interviews, participants reported little to no difficulty in navigating through the app and understanding the instructions. Participants in the second round preferred the smartphone over the tablet. There were 3 participants who preferred the smartphone more because it was easier to hold against their chest. The tablet was preferred by 1 participant because it was easier to read, while 1 reported no differences. No other problems related to ease of use were identified during the second round of interviews.

##### Perceived Usefulness

Similar to responses from the first interviews, 4 participants reported a benefit of learning about their risk of falling. Participants also reported that Steady would be useful for them and other older adults, especially for those who do not have access to fall risk screening.

In this place [retirement community] we have a lot of exercises and programs and balance tests, but I think there are a lot of people who are at home and who don’t have access to all that, and I think it could be very helpful to them.

Female, 83 years old

After receiving their fall risk score, 4 participants also wanted to understand fall prevention strategies they could adopt. Participants suggested the app displaying simple exercises that they could practice at home.

Furthermore, not only did participants find Steady to be useful but they also reported that they would be able to use it on their own. Of the 5 participants, 4 reported that they could complete the app on their own. Because the tests are short, the instructions are clear, and the app is easy to follow, participants reported that they would be able to use Steady in their own homes. There was 1 participant who indicated they would want a caregiver’s help to assist in spotting during the balance tasks.

The instructions were clear and I could download this on my own phone and use it on my own. [Male, 85 years old].

#### Usability Testing Questionnaire

The average score for the SUS on the smartphone was 84, and the average score for the SUS on the tablet was 80 (Figure 9). The average scores on each of the 10 items are reported in Table 3.

## Discussion

### Principal Findings

The purpose of this study was to develop a mobile app to measure fall risk in older adults and to test whether the app was usable by an older adult population. The iterative development and testing of a fall risk mobile app resulted in a usable device for older adults to measure their risk of falling. Through 2 rounds of usability testing, Steady accommodated for age-related perceptual, cognitive, and motor changes to promote use in older adults. When using Steady, participants reported that the app was easy to follow, they had little difficulty receiving a fall risk score (ie, positive ease of use), and the app was beneficial to bring awareness and knowledge of their risk of falling (ie, high perceived usefulness). High SUS scores also indicated high usability for both the smartphone and tablet, although older adults appeared to prefer using the phone for the balance activities.

This study suggests the potential for mobile technology to offer fall risk screening to older adults. The 2 themes generated from the interviews were perceived ease of use and perceived usefulness. Both perceived ease of use and perceived usefulness are factors that predict technology acceptance among older adults [5]. This suggests that older adults may have high acceptance of a fall risk app. Furthermore, a fall risk app that older adults find both usable and useful has high potential to provide falls screening to older adults outside a clinical setting. Along with previous studies that have found smartphones to provide valid balance and fall risk screening, mobile technology may offer a solution to identify high fall risk older adults to seek fall prevention treatment.

Through iterative usability testing, we identified key lessons to use when developing a mobile health (mHealth) app for older adults. First, instructions should be as clear and simple as possible. This became evident when the 30-second balance task instructions were confusing for our participants. Rewording the instructions and maintaining the consistency of these instructions drastically improved performance. Second, measuring fall risk is a necessary step to prevent falls, but older adults also wanted to learn how to lower their fall risk. While participants reported high usefulness, there is potential to increase usefulness by adding prevention strategies. When developing a health app, both measurement and prevention strategies should be taken into consideration. Third, a smartphone can be just as effective as a tablet if the app has high perceived ease of use. We found that by incorporating large font sizes, keeping text consistent, and using contrasting colors, participants found no differences in reading or entering information in the phone or tablet. Because Steady involves holding the device to the chest, the smartphone was found to be the more feasible device. Following these lessons may help develop a highly usable mHealth app for older adults.

### Comparison With Prior Work

To the authors’ knowledge, this is the first study to test the usability of an app that measures fall risk in older adults. Because fall risk screening is underutilized in clinical settings, this study suggests that a smartphone app can not only offer fall risk screening but also be used by older adults. Compared to previous studies, Steady provides a quick and understandable fall risk output. A previous study used a smartphone worn at the hip to monitor fall risk during a dancing game [28]. Another study tested the usability of mobile technology to detect falls in older adults [29]. Although both studies found high usability, neither the game nor the fall detection app provided a fall risk score for users. Providing fall risk knowledge to older adults is the first step to seeking treatments to lower their risk of falling.

A previous study developed and tested an app to measure fall risk based on the Aachen Fall Prevention Scale [15]. This app determines fall risk based on a set of questionnaires and completion of a 10-second balance task. Similarly, the Steady app also includes questionnaires and mobility tasks, but it utilizes valid and known predictors of fall risk and incorporates 5 balance tasks that are related to elevated fall risk in older adults. Furthermore, the previous study did not test the usability, and it is unclear whether older adults found the app easy to use on their own.

A recent study performed a survey of German-speaking internet users to identify features of a falls prevention app would increase user satisfaction, such as having fall risk treatments decided by a health care professional or having gaming elements incorporated for physical activity integration [30]. Steady accomplishes 1 of their findings, namely, to identify fall risk through a standardized test [30]. Because Steady is a fall risk identification app and currently does not include an intervention component, the other features do not apply. Future studies should determine whether the design features reported from the survey are consistent with those identified in the United States, and future iterations of fall risk assessment and prevention apps should follow these guidelines.

### Limitations

A limitation in the design of Steady is that the balance tasks are constrained to individuals who can stand with or without aid. This limits older adults with significant mobility impairments (ie, wheelchair users) from using the app. Wheelchair users have shown to have an elevated fall risk [28], and future iterations of Steady should develop and test fall risk for wheelchair users. Steady also uses visual text to guide users through the app, which is a limitation for older adults with significant vision impairment. Future iterations of Steady should also include an auditory instruction to guide users through the app. It is also possible that individuals with pacemakers may find that holding a smartphone or tablet to their chest interferes with their pacemaker. Therefore, future efforts should also determine whether the phone can be placed at the hip or another area away from a pacemaker.

In addition, our sample of older adults is well-educated. Almost all participants had a college degree or higher. Those with higher education levels may also have greater technology experience, and they may perceive different issues with usability than older adults with lower education levels. Although smartphone and tablet usage were recorded from participants, the type of device (ie, Android, Apple, or Windows), was not recorded. Therefore, it is unclear if preference toward the smartphone was due to current usage of an Android smartphone. Future studies will collect the type of operating platform in addition to technology usage.

While the accelerometer embedded in the smartphone captured data during the balance tasks, the acceleration results were not incorporated into the final fall risk score. In the next iteration, the fall risk score will include both balance performance from acceleration data and health history questionnaire data. This will enhance the classification of low-, moderate-, and high-risk fallers. Furthermore, the next iteration will also include fall prevention strategies, as this was a common request among participants. Future directions should also include a long-term evaluation of Steady and determine its health impact on older adults. Next steps for Steady will be to determine the validity and reliability of the algorithm compared with standard fall risk tests in older adults. Ultimately, a fall risk app that is usable for older adults, valid compared with standard fall risk assessments, and reliable over time may provide a tool to increase knowledge of individual fall risk in older adults.

### Conclusions

In conclusion, through a mixed-methods, iterative design, we developed and tested an app on a smartphone and tablet to measure older adults’ risk of falling. After 1 round of usability testing, confusion with task instructions and visual and tactile errors were corrected. After the second round of testing, older adults found the app useful and easy to use. High SUS scores for both the smartphone and tablet also indicated high usability, but participants preferred holding the smartphone over the tablet. A fall risk app has the potential to be used by older adults to measure their risk of falling.

## Appendix

Multimedia Appendix 1 Questions asked during semistructured interviews for usability testing.

## Abbreviations

mHealth: mobile health
SUS: Systematic Usability Scale
TUG: Timed Up and Go
