# Supplementary material for: A Fall Risk mHealth App for Older Adults: Development and Usability Study
Source: JMIR Aging. 2018 Nov 20;1(2):e11569. doi: 10.2196/11569 (PMC6716481; doi:10.2196/11569)
Supplement: Multimedia Appendix 1 [file aging_v1i2e11569_app1.pdf]

Chart Semi-Structured Interview

The following questions were used to understand participants' thoughts on the feasibility and usability of the fall risk app. For the semi-structured interview, the examiner(s) asked the following questions to the participants and notated responses. Examiners may ask follow-up questions as they see appropriate based on the responses. The interviews were recorded to allow for transcription and further analysis.

1. "What are your first impressions of the app when I handed it to you?"

2. "What do you think about the design and layout of the app?"

3. "What would make this app easier to follow or understand?"

4. "How do you think app would be useful or beneficial to people like you?"

5. What other information do you wish this app can provide you?

6. Do you feel comfortable using this mobile health app yourself or would you want a caregiver's help?
